# BRAF and MEK Inhibitors and Their Toxicities: A Meta-Analysis

**DOI:** 10.3390/cancers15010141

**Published:** 2022-12-26

**Authors:** Mattia Garutti, Melissa Bergnach, Jerry Polesel, Lorenza Palmero, Maria Antonietta Pizzichetta, Fabio Puglisi

**Affiliations:** 1 CRO Aviano, National Cancer Institute, IRCCS, 33081 Aviano, Italy; 2 Department of Medicine, University of Udine, 33100 Udine, Italy; 3 Unit of Cancer Epidemiology, Centro di Riferimento Oncologico di Aviano (CRO) IRCCS, 33081 Aviano, Italy; 4 Department of Dermatology, University of Trieste, 34123 Trieste, Italy

**Keywords:** BRAF, MEK, BRAF inhibitors, MEK inhibitors, meta-analysis, toxicities, melanoma, cancer

## Abstract

**Simple Summary:**

To date, one of the reference therapies for the treatment of mutated BRAF metastatic melanoma is based on the combination of BRAF and MEK inhibitors. Although many trials have compared BRAF and MEK inhibitor combination therapies, there is no evidence of superiority in the use of one of the three combinations over the others. Furthermore, comparative data on safety are scarce. To help clinicians tailor patients’ treatment using the most appropriate BRAF/MEK inhibitor combinations, we performed a meta-analysis of adverse events associated with each treatment combination.

**Abstract:**

Purpose: This meta-analysis summarizes the incidence of treatment-related adverse events (AE) of BRAFi and MEKi. Methods: A systematic search of Medline/PubMed was conducted to identify suitable articles published in English up to 31 December 2021. The primary outcomes were profiles for all-grade and grade 3 or higher treatment-related AEs, and the analysis of single side effects belonging to both categories. Results: The overall incidence of treatment-related all-grade Aes was 99% for Encorafenib (95% CI: 0.97–1.00) and 97% for Trametinib (95% CI: 0.92–0.99; *I*^2^ = 66%) and Binimetinib (95% CI: 0.94–0.99; *I*^2^ = 0%). In combined therapies, the rate was 98% for both Vemurafenib + Cobimetinib (95% CI: 0.96–0.99; *I*^2^ = 77%) and Encorafenib + Binimetinib (95% CI: 0.96–1.00). Grade 3 or higher adverse events were reported in 69% of cases for Binimetinib (95% CI: 0.50–0.84; *I*^2^ = 71%), 68% for Encorafenib (95% CI: 0.61–0.74), and 72% for Vemurafenib + Cobimetinib (95% CI: 0.65–0.79; *I*^2^ = 84%). The most common grade 1–2 AEs were pyrexia (43%) and fatigue (28%) for Dabrafenib + Trametinib and diarrhea for both Vemurafenib + Cobimetinib (52%) and Encorafenib + Binimetinib (34%). The most common AEs of grade 3 or higher were pyrexia, rash, and hypertension for Dabrafenib + Trametinib (6%), rash and hypertension for Encorafenib + Binimetinib (6%), and increased AST and ALT for Vemurafenib + Cobimetinib (10%). Conclusions: Our study provides comprehensive data on treatment-related adverse events of BRAFi and MEKi combination therapies, showing related toxicity profiles to offer a helpful tool for clinicians in the choice of therapy.

## 1. Introduction

In the early 1980s, new discoveries in the fields of molecular biology, cell biology, and immunology led to a better understanding of the mechanisms underlying the neoplastic transformations in cells [1,2].

These discoveries opened the doors to the targeted therapy, which is based on small selective inhibitory molecules or monoclonal antibodies that make it possible to act on a specific target expressed by cancer cells and not by healthy ones, allowing to significantly reduce toxicity and side effects [1,2]. The target therapy spectrum includes chemical small molecules such as intracytoplasmic serine/threonine kinase inhibitors, which include BRAF inhibitors (Vemurafenib, Dabrafenib, and Encorafenib) and MEK inhibitors (Trametinib, Cobimetinib, and Binimetinib) [2]. The role of these drugs, demonstrated by their biological target (BRAF or MEK), is closely related to the MAPK pathway, a cascade involved in extracellular signal transduction to the nucleus, promoting the expression of genes with a central role in cell proliferation, differentiation, survival, and apoptosis [3,4,5,6,7].

In clinical practice, the prominence of this effect is mainly ascribable to melanoma treatment; in fact, in about 40–60% of patients affected by cutaneous melanoma, tumor cells harbor a specific mutation called BRAF-V600E, which results as targetable by BRAF inhibitors which interfere with this signal transduction pathway, causing apoptosis in melanoma cells [3,8,9].

The rationale behind the use of the combination of BRAF and MEK inhibitors is to prevent the aberrant activation of mutated BRAF in the context of the RAF-MEK-ERK pathway [4,7,10,11,12,13]. The BRAF inhibitors demonstrated a greater efficacy when compared to standard chemotherapy, improving the outcome of patients in terms of both progression-free and overall survival; however, the benefit was transient, with median progression-free survival ranging between 6 and 7 months [14,15,16]. Furthermore, BRAF inhibitors, by paradoxically activating the molecular pathway of MAP kinases, favored the development of secondary tumors, especially cutaneous squamous cell carcinomas, and the main cause of monotherapy failure was represented by acquisition of resistance mechanisms, mainly due to a reactivation of the MAP kinase pathway [17,18,19].

To overcome said issue, in the reference therapy for BRAF-mutated metastatic melanoma, BRAF inhibitors are used in combination with MEK inhibitors to act simultaneously against two different targets of the same pathway, evading the cancerous cell-intrinsic resistance [3,8,9,20]. MEK inhibitors, in fact, block the paradoxical activation of the MAP kinase pathway and do not display the criticalities previously noted following the use of BRAF inhibitors [11,12].

Currently, three different doublets are available in clinical practice (Dabrafenib plus Trametinib, Vemurafenib plus Cobimentinib, and Encorafenib plus Binimetinib), and each one of them is present in at least one phase 3 clinical trial [14,21,22,23]. However, there are no studies involving a direct comparison between the various BRAF and MEK inhibitors, meaning there are no evident signs of superiority in the use of one combination over the others, especially in terms of safety [11,12,17,24].

To help clinicians to better select the right BRAF/MEK inhibitor combinations for the right patients, we performed a meta-analysis with the aim to indirectly compare the different safety profiles of BRAF and MEK inhibitors. With these data, we have also elaborated a tool to facilitate the assignment of drugs to the most frequently encountered clinical scenarios.

## 2. Materials and Methods

### 2.1. Search Strategy and Selection Criteria

One database—Medline (PubMed)—was used to search for relevant articles published in English in a peer-reviewed journal, with the last search update on 31 December 2021. Systematic research was set up to identify published studies on BRAF and MEK inhibitor therapies that reported treatment-related adverse events, filtering only English articles obeying this query: “LX-4032 OR PLX4032 OR RG-7204 OR RG7204 OR RO-51-85426 OR RO-5185426 OR RO5185426 OR Vemurafenib OR Zelboraf OR GDC 0973 OR GDC-0973 OR GDC0973 OR RG 7420 OR RG-7420 OR RG7420 OR RO5514041 OR XL 518 OR XL-518 OR XL518 OR Cobimetinib OR Cotellic OR GSK-2118436 OR GSK-2118436A OR GSK2118436 OR GSK2118436A OR Dabrafenib OR Tafinlar OR GSK 1120212 OR GSK-1120212 OR GSK1120212 OR JTP 74057 OR JTP-74057 OR Trametinib OR Mekinist OR LGX-818 OR LGX818 OR NVP-LGX-818-NXA OR NVP-LGX818 OR NVP-LGX818-NXA OR Encorafenib OR Braftovi OR ARRY-162 OR ARRY-438162 OR MEK-162 OR MEK162 OR NVP-MEK162 OR Binimetinib OR Mektovi”.

The selection of the studies was conducted based on the following inclusion criteria: (a) studies on BRAF inhibitors and MEK inhibitors, (b) studies with a sample size of 20 patients or higher, and (c) studies reporting data on toxicity related to BRAF and MEK inhibitors. Reviews, case reports, and studies on the evaluation of QoL (quality of life) were excluded. The retrieved articles were screened and selected by two independent authors (M.B. and L.P.) according to the inclusion and exclusion criteria, and disagreements were settled by a third researcher (M.G.). This process resulted in 91 eligible records (Table S1) [9,14,16,21,22,23,25,26,27,28,29,30,31,32,33,34,35,36,37,38,39,40,41,42,43,44,45,46,47,48,49,50,51,52,53,54,55,56,57,58,59,60,61,62,63,64,65,66,67,68,69,70,71,72,73,74,75,76,77,78,79,80,81,82,83,84,85,86,87,88,89,90,91,92,93,94,95,96,97,98,99,100,101,102,103,104,105,106,107,108,109]. Among them, 68 are about melanoma and 23 are about other cancers. Line of therapy was not a selection criterion.

Quality assessment and risk of bias were evaluated through the ROBINS-I tool [110]. All included articles reported low risk.

Adverse events were graded according to the National Cancer Institute Common Terminology Criteria for Adverse Events (CTC-AE) version 4.0.

### 2.2. Data Extraction

From each included study, the parameters that were taken into consideration were: author, year of publication, study design, country, period of conduction, total number of patients, and number of patients reporting adverse events according to grade. 

### 2.3. Statistical Analysis

The pooled proportion of adverse events and corresponding 95% confidence interval (CI) were calculated according to random-effects models of DerSimonian and Laird [111], using the logit transformation. Statistical heterogeneity among studies was evaluated using the *I*^2^ and τ^2^ statistics. Publication bias was assessed through a funnel plot [112]. Influence analysis was performed by calculating the pooled proportion, omitting one study at a time.

The results of the meta-analysis were presented graphically using forest plots, plotting the individual papers, pooled proportions, and corresponding 95% CI. Analyses were conducted using R 4.1, and statistical significance was defined as *p* < 0.05 (two-sided).

The proportion of adverse events was then correlated to oncological outcome with an ecological approach, calculating the Spearman correlation coefficient (r). 

## 3. Results

**Figure 1:**
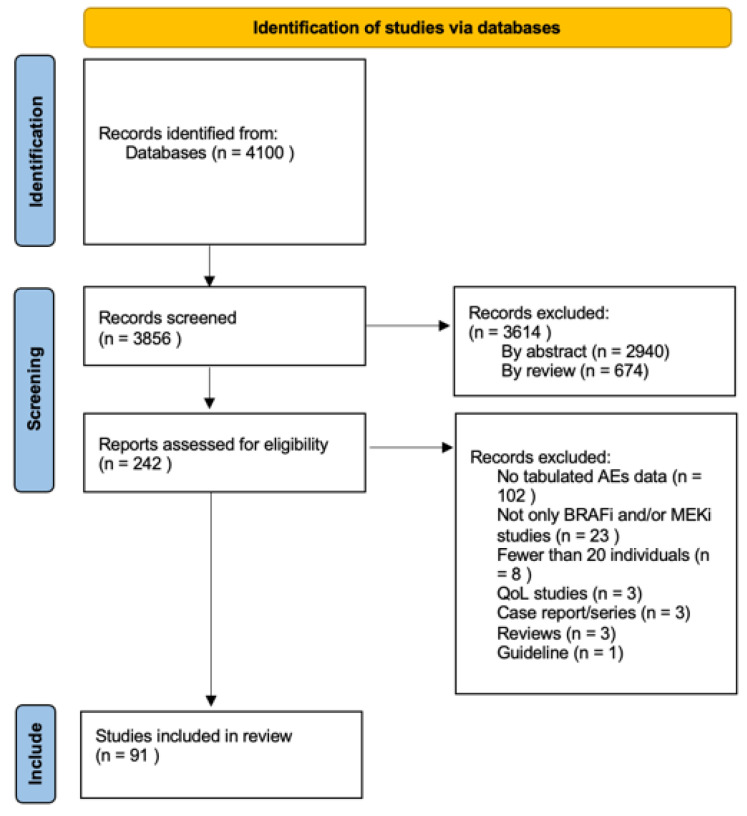
PRISMA flow chart of study inclusion process [113].

Our initial research, performed on PubMed publications, identified 3856 records, of which only 91 were deemed eligible, involving a total of about 23,000 patients (Figure 1). The 91 studies that satisfied the inclusion criteria were analyzed (Table S1) [9,14,16,21,22,23,25,26,27,28,29,30,31,32,33,34,35,36,37,38,39,40,41,42,43,44,45,46,47,48,49,50,51,52,53,54,55,56,57,58,59,60,61,62,63,64,65,66,67,68,69,70,71,72,73,74,75,76,77,78,79,80,81,82,83,84,85,86,87,88,89,90,91,92,93,94,95,96,97,98,99,100,101,102,103,104,105,106,107,108,109].

A detailed evaluation of the risk of bias for each study reported a low level of risk for all included studies (Table S2) [110].

### 3.1. Any-Grade Toxicity

**Figure 2:**
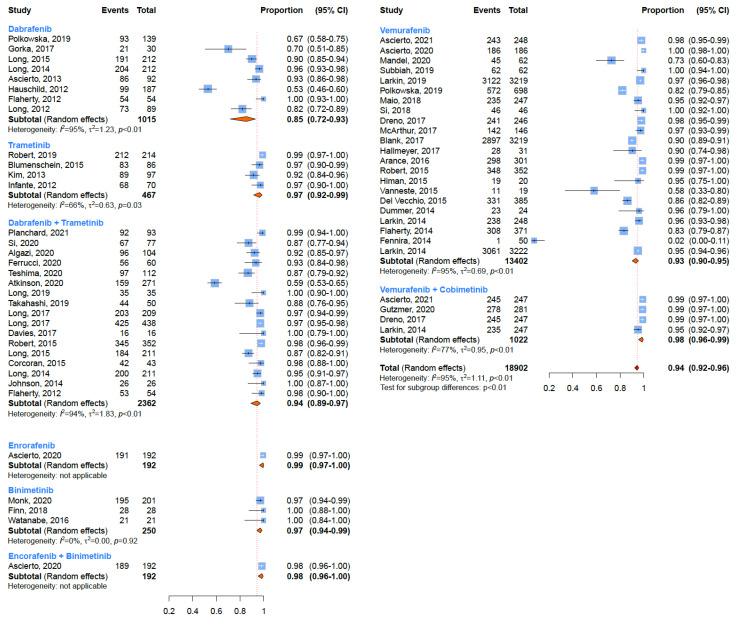
Overall incidences of all-grade adverse events according to treatment regimen.

Adverse events of any grade (G1–G5) have been registered in a large percentage of patients treated with BRAF and MEK inhibitors (Figure 2). Among BRAF inhibitors, Vemurafenib induced adverse events of any grade in 93% of patients (95% CI: 90–95%), Dabrafenib in 85% (95% CI: 72–93%), whilst patients who underwent treatment with Encorafenib developed a toxicity of any grade in 99% of cases (95% CI: 97–100%) (Table 1). Regarding MEK inhibitors, 97% of patients who underwent treatment with Trametinib (95% CI: 92–99%) and Binimetinib (95% CI: 94–99%) developed a toxicity of any grade (Table 1). As for the combined therapies, Vemurafenib plus Cobimetinib induced side effects of any grade in 98% of patients (95% CI: 96–99%) (Table 1). This kind of toxicity was reported in 94% of patients who, instead, underwent treatment with an association of Dabrafenib and Trametinib (95% CI: 89–97%) and in 98% of patients who underwent treatment with an association of Encorafenib plus Binimetinib (95% CI: 96–100%) (Table 1). Significant heterogeneity (*p* < 0.05) emerged for all considered treatments.

### 3.2. Grade 3 or Higher Toxicity

**Figure 3:**
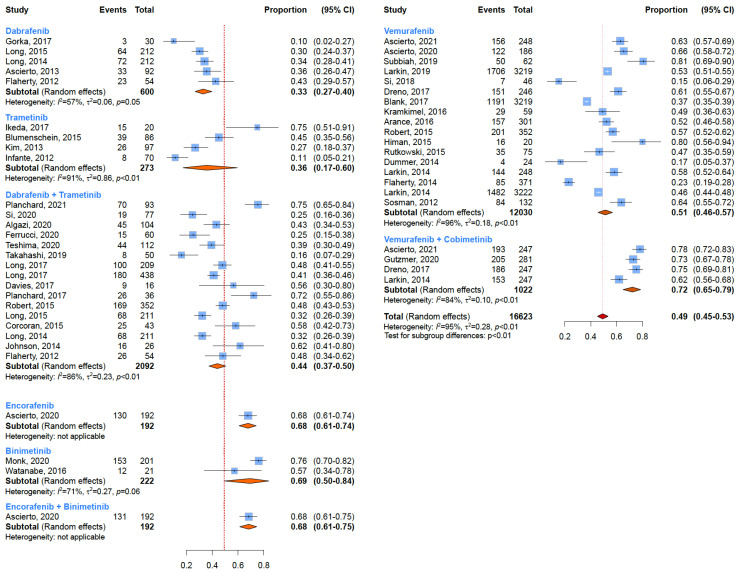
Overall incidences of all-grade adverse events for every treatment regimen.

Focusing on BRAF inhibitors (Figure 3), induced side effects of grade 3–5 were reported more frequently among patients undergoing treatment with Encorafenib (68%; 95% CI: 61–74%) and Vemurafenib (51%; 95% CI: 46–57%) than those treated with Dabrafenib (33%; 95% CI: 27–40%) (Table 1). Among MEK inhibitors, induced side effects of grade 3–5 were higher for Binimetinib (69%; 95% CI: 50–84%) than for Trametinib (36%; 95% CI: 17–60%) (Table 1). As for the combined therapies, induced side effects of grade 3–5 were reported by 72% (95% CI: 65–79%) of patients undergoing Vemurafenib plus Cobimetinib and in 68% of patients treated with Encorafenib plus Binimetinib (95% CI: 61–75%). Only 44% (95% CI: 37–50%) of patients undergoing treatment with the association of Dabrafenib and Trametinib developed a grade 3–5 toxicity (Table 1).

### 3.3. G1–G2 Adverse Events

When analyzing the data on single G1–G2 adverse events, there is a variability in the incidence of the various side effects considered.

As for monotherapy, Vemurafenib is associated with arthralgia in 40% of cases, rash in 34%, and fatigue in 33%, while Dabrafenib causes fatigue in 41% of patients, pyrexia in 32%, and rash in 31%. Significant incidence data above 30% were also observed for Trametinib causing rash in 38% of cases, Encorafenib associated with alopecia in 56%, and Binimetinib causing rash in 64%, diarrhea in 40%, and vomiting in 38%.

**Figure 4:**
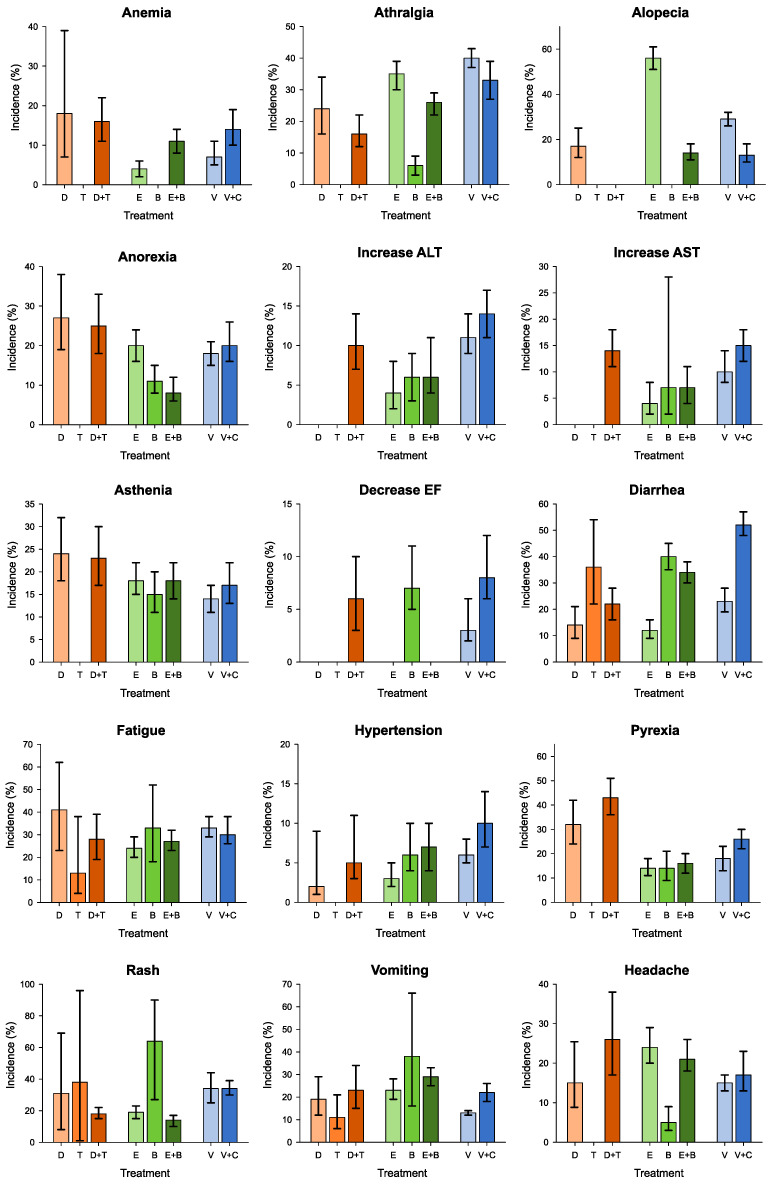
Summary estimates of incidence (%) and corresponding 95% confidence interval of grade 1–2 adverse events according to treatment. ALT: alanine aminotransferase; AST: aspartate aminotransferase; C: Cobimetinib; D: Dabrafenib; E: Encorafenib; EF: ejection fraction; T: Trametinib; V: Vemurafenib.

As for the combination therapy, Vemurafenib plus Cobimetinib causes diarrhea in 52% of cases, Encorafenib plus Binimetinib causes diarrhea in 34%, and finally, Dabrafenib and Trametinib are associated with pyrexia in 43% (Figure 4).

### 3.4. G3–G5 Adverse Events

When analyzing the data involving single G3–G5 adverse events, regarding monotherapy, the incidence is equal to or less than 5% in most cases, such as: increase in transaminases and asthenia, and decreased ejection fraction, diarrhea, and fatigue.

Higher rates of incidence are instead recorded in the course of therapy with Encorafenib, which induces arthralgia in 13% (95% CI: 0.05–0.27) of patients, Trametinib causes hypertension (95% CI: 0.08–0.16) and skin rash (95% CI: 0.08–0.16) in 11% of cases, and Binimetinib induces anemia in 8% (95% CI: 0.01–0.41, *p*-value < 0.01) of cases.

**Figure 5:**
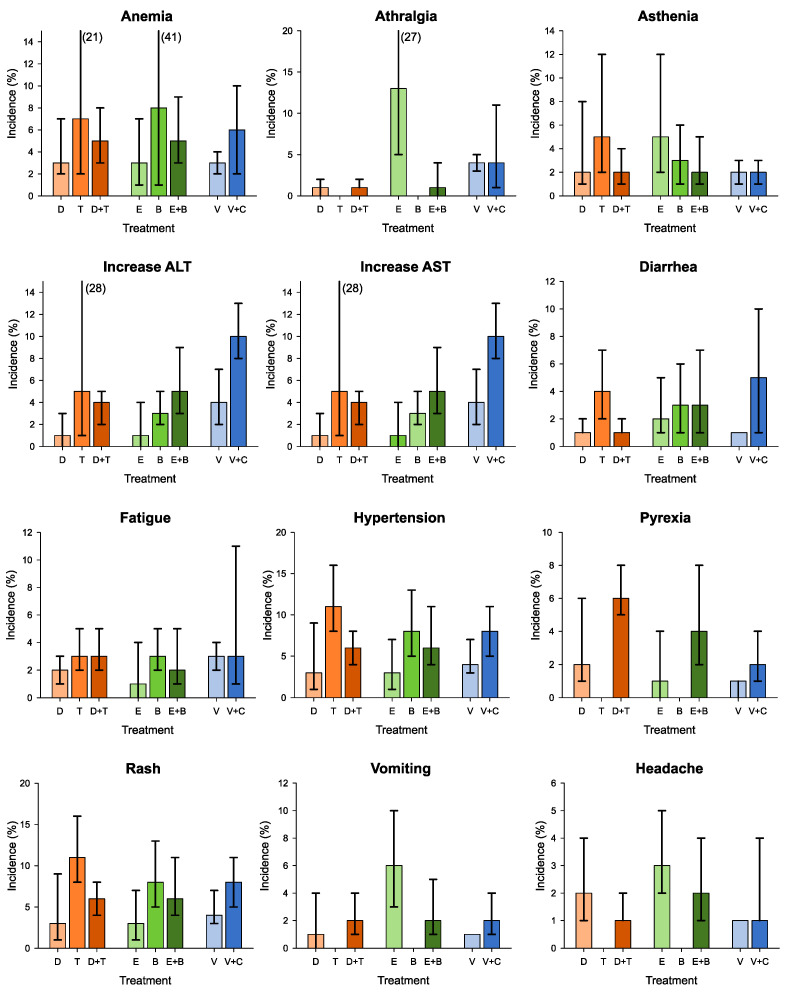
Summary estimates of incidence (%) and corresponding 95% confidence interval of adverse events of grade 3 or higher according to treatment. ALT: alanine aminotransferase; AST: aspartate aminotransferase; C: Cobimetinib; D: Dabrafenib; E: Encorafenib; EF: ejection fraction; T: Trametinib; V: Vemurafenib.

Regarding the combination therapy, Vemurafenib plus Cobimetinib represents the pharmacological doublet associated with a higher frequency of adverse events, inducing an increase in transaminases in 10% (95% CI: 0.08–0.13) of cases, skin rash in 8% (95% CI: 0.05–0.11), vomiting in 8% (95% CI: 0.01–0.04), and hypertension in 8% (95% CI: 0.05–0.11) (Figure 5).

Grade 5 adverse events were registered in each treatment group. One case of intracranial hemorrhage was registered following treatment with Dabrafenib. Vemurafenib induced a higher number of grade 5 adverse events, the most frequent of which were unknown deaths, general physical health deterioration, cerebral hemorrhage, cerebrovascular accident, pneumonia, and intracranial tumor hemorrhage (Table S3, Figure S1). Two patients treated with Trametinib died of sudden and unknown death, whilst only one patient treated with Binimetinib died of unknown causes. One patient treated with the Encorafenib plus Binimetinib doublet was lost to suicide, and five patients treated with Vemurafenib and Cobimetinib suffered from cardiac arrest, coma, clostridium difficile colitis, myocardial infarction, and pneumonia. Finally, five patients were lost to grade 5 adverse events in the treatment with Dabrafenib and Trametinib: two cerebral hemorrhages, one brain stem hemorrhage, one pneumoperitoneum, and one disease progression (Table S3, Figure S1).

### 3.5. Dose Reduction and Treatment Interruption

**Figure 6:**
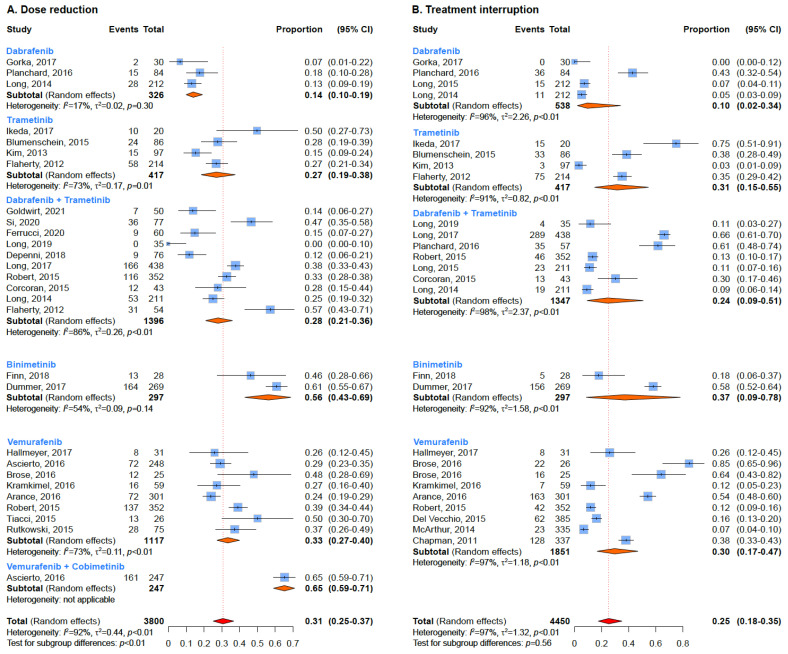
Summary estimates of dose reduction (**A**) and treatment interruption (**B**) according to treatment regimen.

Among BRAF inhibitors, a necessary dose reduction was registered in 33% of patients treated with Vemurafenib (95% CI: 27–40%) and 14% of patients treated with Dabrafenib (95% CI: 10–19%), whilst, when analyzing MEK inhibitors, it was registered in 27% of patients treated with Trametinib (95% CI: 19–38%) and 56% of patients receiving Binimetinib (95% CI: 43–69%). As for the combined therapies, the data show a dose reduction applied to 65% of patients treated with Vemurafenib and Cobimetinib (95% CI: 59–71%), and to 28% of patients treated with Dabrafenib plus Trametinib (95% CI: 21–36%). Treatment was discontinued in 30% of patients treated with Vemurafenib (95% CI: 17–47%) and 10% of patients treated with Dabrafenib (2–34%), whilst among MEK inhibitors, it was interrupted in 31% of patients treated with Trametinib (95% CI: 15–55%) and 37% of patients treated with Binimetinib (95% CI: 9–78%). As for the combined therapies, treatment was discontinued in 24% of patients treated with Dabrafenib plus Trametinib (95% CI: 9–51%) (Figure 6).

### 3.6. Adverse Events and Oncological Outcomes

**Figure 7:**
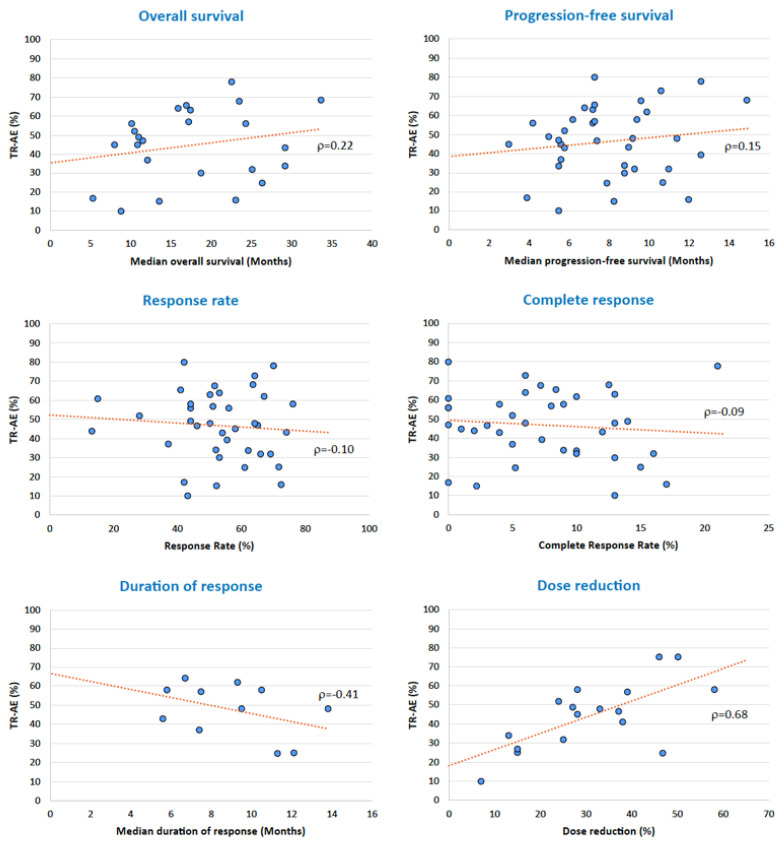
Correlation between incidence of all-grade adverse events and oncological outcomes.

To evaluate whether side effects have an impact on prognosis, we correlated the reported incidence of side effects and some oncological outcomes. No correlation emerged between the incidence of side effects and the median progression-free survival (r = 0.15), response rate (r = −0.10), and complete response rate (r = −0.09). There was a slight positive correlation, instead, between the incidence of side effects and the median overall survival (r = 0.22). A slight but negative correlation was instead registered between the incidence of side effects and the median duration of response (r = −0.41). Finally, there was a strong, direct, positive correlation between the incidence of side effects and dose reduction (r = 0.68) (Figure 7).

## 4. Discussion

This meta-analysis, through the review of toxicity profiles of BRAF and MEK inhibitor therapies, both in monotherapy and in combined regimes, aimed to find the most suitable doublet for the individual patient.

BRAF inhibitors are selective inhibitors of BRAF kinase with immunomodulatory action. The toxicity profiles amongst them are similar: rash, joint pain, and fatigue are recorded with overlapping incidence rates, but may present a few exceptions, such as the excessive sensitivity to sunlight caused by Vemurafenib, or hyperkeratosis or dysesthesia seem to occur more frequently, particularly with Encorafenib monotherapy [17,18,114,115]. Being inhibitors to the BRAF kinase also means they have a common feature biologically related to the paradoxical activation of the MAP kinase pathway associated with upstream activation of pre-existing RAS mutations in a specific type of cell: keratinocytes; in fact, a frequent adverse event related to the use of BRAF inhibitors is the development of secondary cutaneous squamous cell carcinomas and keratoacanthomas [105,116,117].

MEK inhibitors, on the other hand, have extrinsic activity against MEK1/2 kinases involved in the MAPK pathway [15,118,119]. Results on adverse events display that rash, diarrhea, peripheral edema, and fatigue are common to all three drugs belonging to this class [16,25]. On the other hand, acneiform dermatitis and papulopustular rash are specifically related to Trametinib assumptions, just as nausea and vomiting are characteristically recorded following therapy with Binimetinib [15,107].

The end purpose is to be able to tailor a therapy, avoiding specific side effects in patients that already suffer from mostly chronic diseases involving the same organs or apparatuses. Following this statement, the significance of single side effects is not considered in absolute value but in relation to the clinical conditions and comorbidities of the patient on the receiving end of the treatment.

To obtain this and achieve accurate results with a complete and clear profile of side effect incidence rates, the analysis has been divided into four different fields: two of which focus on any-grade and grade 3 through 5 side effects, and two other fields concentrating specifically on single side effects belonging to both previously mentioned categories. The side effects we chose to analyze singularly are the ones deemed both common and clinically relevant, and they include: anemia, arthralgia, alopecia, asthenia, diarrhea, fatigue, pyrexia, rash, vomiting, headache, transaminase level increases, anorexia, hypertension, and decreased ejection fraction.

Although a direct comparison between the three drug combinations is not possible, the incidence rates of any-grade side effects appear to be comparable and higher than 90% for all 3 doublets, whilst a greater divergence is registered when focusing on grades 3 through 5 side effects. Specifically, the results highlight a higher incidence of the latter following treatment with Vemurafenib plus Cobimetinib and Encorafenib plus Binimetinib, where values stand, respectively, at 72% and 68%, whilst the remaining doublet, Dabrafenib plus Trametinib standing at 44%, shows remarkably lower incidence rates. This implies that whilst lower-grade side effects are expected no matter which doublet is chosen for therapy, we can anticipate a significantly decreased rate of high-grade side effects when choosing the Dabrafenib and Trametinib doublet. 

In regard to the analysis of lower-grade side effects (G1 and G2), the results describe that systemic side effects such as arthralgia, alopecia, asthenia, fatigue, rash, headache, and pyrexia are frequent in all three therapeutic regimens, with small variations: the combination of Vemurafenib and Cobimetinib presents higher incidence rates of arthralgia, fatigue, and skin rash; on the other hand, the onset of headache, pyrexia, and asthenia are more frequently caused by the combination of Dabrafenib and Trametinib. Analyzing specific organ side effects, the main toxicity involves the gastrointestinal system: greater than 20% in all three drug combinations for both diarrhea and vomiting. Specifically, the highest incidence of G1–G2 diarrhea, 52%, is registered in the combination therapy with Vemurafenib and Cobimetinib, while the highest incidence of vomiting, 29%, is observed following the use of Encorafenib and Binimetinib. Intermediate incidence rates ranging from 8% (Encorafenib plus Binimetinib) to 25% (Dabrafenib plus Trametinib) are recorded for anorexia and ranging from around 7% (Encorafenib plus Binimetinib) to approximately 15% (Vemurafenib plus Cobimetinib) for liver toxicity, with increased transaminase levels. 

Lastly, incidence rates lower than 10% were associated with cardiovascular involvement. 

These data show that even though there is a comparable toxicity spectrum when considering systemic side effects, a higher variability is registered between the incidence rates of side effects targeting specific organs or apparatuses. This trend is also noticeable when we switch the focus to higher-grade adverse events.

Some of the grade 3 through 5 side effects present superimposable incidence rates between the three doublets, and these are anemia (5–6%), asthenia (3%), decreased ejection fraction (2–3%), and headache (1–2%). The key factor, though, on which this analysis shines the light, is the unevenness between the doublets. When considering therapies with Dabrafenib plus Trametinib and Encorafenib plus Binmetinib, arthralgia is registered in 1% of cases, rising to 4% when the treatment considered is Vemurafenib plus Cobimetinib. This consideration can be meaningful in orienting towards the therapeutic choice when patients undergoing this specific oncological treatment suffer from chronic joint issues or concomitant rheumatological pathologies.

Hypertension is recorded in 6% of patients treated with Dabrafenib plus Trametinib and with Encorafenib plus Binimetinib, an incidence rate that increases to 8% when considering the Vemurafenib and Cobimetinib doublet. In this case, the first two therapeutic options should be favored. Even if the difference appears to be small, granting a better cardiovascular toxicity profile in a general population commonly affected by these kinds of comorbidities is preferable. On the same line, pyrexia is registered in 2% of cases in the Vemurafenib and Cobimetinib combined therapy, an incidence that increases to 4% when we consider Encorafenib plus Binimetinib and reaches 6% in case of treatment with Dabrafenib plus Trametinib. The difference between these numbers in absolute value can seem small, but when considering the comparison regardless of what the actual number is, what is clear is that Dabrafenib plus Trametinib leads to an essentially tripled incidence rate of pyrexia, a factor that should be kept in mind when choosing the appropriate doublet.

When it comes to gastrointestinal side effects, both vomiting and diarrhea present higher incidence rates following therapy with Vemurafenib and Cobimetinib, respectively, 8% and 5%, which decreases for the other doublets: vomiting rates diminish to 2% in both the other doublets, and diarrhea rates decrease to 1% and 3%, respectively, for Dabrafenib plus Trametinib and Encorafenib plus Binimetinib. Therefore, although our data are descriptive in nature, it could be speculated that Dabrafenib and Trametinib, compared to other drug doublets, might induce gastroenteric grade 3–5 side effects in a lower percentage of cases.

Vemurafenib plus Cobimetinib is a doublet that also results in a relevant rate of side effects such as skin rash (8%) and hepatotoxicity. In fact, it determines an increase in hepatic cytonecrosis indices (AST and ALT) in 10% of cases, compared to essentially halved rates if we instead consider the therapies with Dabrafenib and Trametinib (4%) and with Encorafenib and Binimetinib (5%). This information is relevant when we consider hepatopathic patients for treatment: the doublets consisting of Dabrafenib plus Trametinib or Encorafenib plus Binimetinib may be preferred over Vemurafenib plus Cobimetinib.

Although the combination of Vemurafenib and Cobimetinib is associated with higher incidence rates of diarrhea compared to other doublets, the same result does not emerge when considering vomiting and anorexia. In fact, although the results are clear in stating that the use of Dabrafenib and Trametinib is associated with a higher incidence rate of anorexia, the same cannot be stated for vomiting, where the data are conflicting. A higher incidence rate of grade 1–2 vomiting is related to the use of Encorafenib and Binimetinib, whilst when it comes to grades 3 or higher toxicity, the V-C doublet emerges. Comparing the results obtained on the incidence profiles of the single most frequent side effects following the prescription of BRAF and MEK inhibitors, it is observed that the combination therapy with Vemurafenib and Cobimetinib is associated with a higher rate of diarrhea, arthralgia, and skin rash for any considered grade. This combination is also correlated to an onset of liver toxicity with a transaminase level increase, and cardiovascular toxicity, resulting in higher incidence rates of hypertension and decreased ejection fraction when compared to the other doublets, even though, focusing especially on the grade 3 or higher decrease in ejection fraction, higher incidence rates were reported in the Dabrafenib and Trametinib therapy regimen. 

In conclusion, based on the results of this meta-analysis, it appears that some doublets may be more suitable than others in certain patient categories in virtue of a lower rate of specific side effects. This study shows that when the chosen combination is Vemurafenib and Cobimetinib, there is a higher incidence of hepatotoxicity; consequently, the use of other doublets presenting the lowest rates of hepatotoxicity could be preferred, in this case the Encorafenib and Binimetinib one. The data are relevant, especially in the absence of a direct comparison, when we consider treatment in patients with underlying liver cirrhosis or rare genetic liver dysfunctions. The same type of reasoning can be applied to cardiovascular diseases. Considering the high incidence of arterial hypertension, heart failure, and atrial and ventricular arrhythmias in the general population, the lower incidence of side effects that could affect the heart should guide the choice of which doublet to prescribe. The lowest incidence rate for increased blood pressure and decreased ejection fraction was recorded in relation to Encorafenib and Binimetinib, potentially becoming the preferred doublet in this specific subset of patients. Considering the progressive trend towards a longer life expectancy, and therefore an increase in numbers of the elderly population, it is crucial to consider side effects such as arthralgia in specific contexts, such as patients suffering from aging-linked arthrosis, chronic arthropathies, and above all, rheumatological diseases that afflict bones and cartilage, such as rheumatoid arthritis. This study shows that treatment with Vemurafenib and Cobimetinib is related to a higher incidence of any-grade arthralgia; therefore, one could move towards the use of the other two doublets, reporting lower and almost overlapping incidence rates.

Furthermore, it was found that combination therapy with Vemurafenib and Cobimetinib shows a higher incidence of any-grade skin rash. In this case, resorting to the use of Dabrafenib and Trametinib or Encorafenib and Binimetinib would represent the better option for patients suffering from dermatological diseases. 

Finally, the main hematological side effect studied was anemia. In this regard, the data are conflicting: Vemurafenib and Cobimetinib, when combined, would determine a higher incidence of grade 3 or higher anemia, whilst a higher incidence of grade 1–2 anemia is associated with the use of Dabrafenib and Trametinib.

The toxicity profile review performed, in addition and relation to patients’ clinical conditions and comorbidities, can prove useful in selecting the best doublet. This analysis, however, presents some limitations. Firstly, even though the amount of gathered data was high and this work turned out to be solid, a direct comparison between drugs was not possible. Furthermore, the non-inclusion of data belonging to either quality of life reports (QoL) or patient-reported outcome measures (PROM) represents another limitation. Nevertheless, the results of this meta-analysis can represent a valuable basis for future studies aimed to identify the optimal doublet to use in a specific clinical context and to define differential toxicity based on cancer type, line of therapy, and baseline biomarkers (e.g., LDH).

## 5. Conclusions

**Figure 8:**
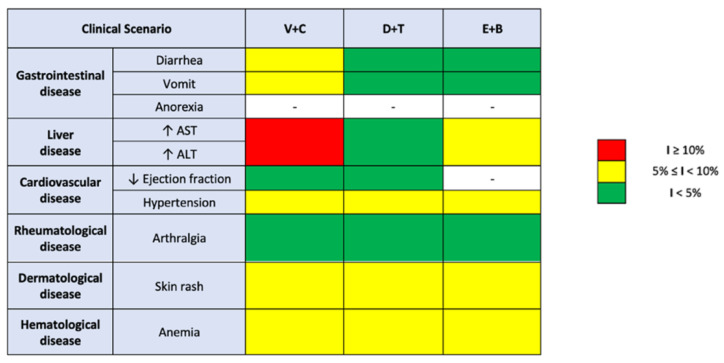
Therapeutic hypotheses amongst the three pharmacological doublets (V + C: Vemurafenib plus Cobimetinib; D + T: Dabrafenib plus Trametinib; E + B: Encorafenib plus Binimetinib) under analysis, considering the clinical scenario and the grade 3–5 side effects’ incidence (I).

The goal of this meta-analysis was to provide a useful tool to guide the clinician towards the best therapeutic option among the different combinations of BRAF and MEK inhibitors used in melanoma treatment (Figure 8). In virtue of that fact, as mentioned earlier, to date, the choice between one of the three combinations is essentially arbitrary. In view of this, our study provided comprehensive data on treatment-related adverse events in BRAF and MEK inhibitor combination therapies, displaying the toxicity profiles of BRAF inhibitors, MEK inhibitors, and combination therapies, providing a valuable tool to guide clinicians in the choice of the optimal therapeutic option for patients with BRAF-mutant melanoma.

## Figures and Tables

**Table 1:** Overall incidence of any grade and G3–G5 adverse events according to treatment.

Treatment	Any Grade % (95% CI)	G3–G5 % (95% CI)
Dabrafenib	85% (72–89%)	50% (45–54%)
Trametinib	97% (92–99%)	36% (17–60%)
Dabrafenib + Trametinib	95% (90–98%)	43% (36–50%)
Encorafenib	99% (96–100%)	68% (61–74%)
Binimetinib	97% (94–99%)	72% (62–81%)
Encorafenib + Binimetinib	98% (95–99%)	68% (61–75%)
Vemurafenib	94% (91–96%)	51% (46–57%)
Vemurafenib + Cobimetinib	98% (96–99%)	72% (65–79%)

CI: confidence interval.

## Supplementary Materials

The following supporting information can be downloaded at: https://www.mdpi.com/article/10.3390/cancers15010141/s1, Table S1: The 91 studies included in the meta-analysis. Table S2: Evaluation of the bias risk according to Risk of Bias in Non-randomized Studies of Interventions guidelines (ROBINS I) [110]. Table S3: Adverse events grade 5; Figure S1: Overall incidences of grade 5 adverse events according to treatment regimen.
