# Supplementary material for: BRAF and MEK Inhibitors and Their Toxicities: A Meta-Analysis
Source: Cancers (Basel). 2022 Dec 26;15(1):141. doi: 10.3390/cancers15010141 (PMC9818023; doi:10.3390/cancers15010141)
Supplement: Supplementary file 1 [file cancers-15-00141-s001.zip › cancers-2062700-supplementary.pdf]

Supplementary materials

Table S1-The 91 studies included in the meta-analysis.

Study PMID	Year	Study type (1= retrospective 2=prospective)	Study phase (1,2,3,4, real world)	Study size	Drug
21639808	2011	2	3	337	V
22735384	2012	2	3	187	D
23020132	2012	2	1-2	54	D
	2012	2	1-2	54	DT
	2012	2	1-2	54	DT
22663011	2012	2	3	214	T
22356324	2012	2	2	132	V
23051966	2012	2	2	89	D
	2012	2	2	83	D
22805291	2012	2	1	70	T
22805292	2012	2	1	26	T
23248257	2013	2	2	97	T
23414587	2013	2	2	30	B
	2013	2	2	41	B
23918947	2013	2	2	92	D
24445759	2014	2	4	371	V
24241686	2014	1	real world	50	V
25037139	2014	2	1-2	66	VC
	2014	2	1-2	63	VC
25287827	2014	2	1-2	26	DT
	2014	2	1-2	45	DT
24582505	2014	2	2	3222	V
24508103	2014	2	3	335	V
24295639	2014	2	3	24	V
25265492	2014	2	3	211	DT
	2014	2	3	212	D
25265494	2014	2	3	247	VC
	2014	2	3	248	V
25399551	2015	2	3	352	V
	2015	2	3	352	DT
26037941	2015	2	3	211	DT
	2015	2	3	212	D
26352686	2015	2	2	28	V
	2015	2	2	26	V
26460303	2015	2	2	21	V
25722381	2015	2	2	86	T
25956405	2015	1	-	27	V

26287849	2015	2	2	20	V
26392102	2015	2	2	43	DT

Study PMID	Year	Study type (1= retrospective 2=prospective)	Study phase (1,2,3,4, real world)	Study size	Drug
24661317	2015	2	real world	19	V
25952781	2015	2	real world	385	V
26557775	2015	2	real world	75	V
27080216	2016	2	2	84	D
27283860	2016	2	2	57	DT
27460442	2016	2	2	26	V
	2016	2	2	25	V
26208946	2016	2	real world	59	V
26981153	2016	2	real world	40	D
26983408	2016	2	3	301	V
27071922	2016	2	1	21	B
27470608	2016	1	-	48	DT
27480103	2016	2	3	247	VC
	2016	2	3	248	V
28151720	2017	2	2	20	T
27993793	2017	2	2	146	V
28268064	2017	2	2	25	DT
28592387	2017	2	2	76	DT
	2017	2	2	16	DT
	2017	2	2	16	DT
	2017	2	2	17	DT
28919011	2017	2	2	36	DT
29121415	2017	2	2	20	T
28444112	2017	2	3	246	V
	2017	2	3	247	VC
28475671	2017	2	3	209	DT
	2017	2	3	211	D
28284557	2017	2	3	269	B
28891408	2017	2	3	438	DT
28152546	2017	2	1	44	B
28501764	2017	2	4	3219	V
28567600	2017	1	-	30	D
28611198	2017	2	1	35	E
28963614	2017	1	-	43	V
29285228	2017	2	3	21	D
29076950	2017	2	2	31	V
29384960	2017	1	-	48	D
	2017	1	-	87	DT

Study PMID	Year	Study type (1= retrospective 2=prospective)	Study phase (1,2,3,4, real world)	Study size	Drug
29477665	2018	2	3	247	V
29573941	2018	2	3	192	EB
	2018	2	3	194	E
	2018	2	3	191	V
30219628	2018	2	3	192	EB
	2018	2	3	194	E
	2018	2	3	191	V
30343620	2018	2	3	438	DT
29724167	2018	2	1	46	V
29785570	2018	2	1	28	B
30081673	2018	1	-	76	DT
29438093	2018	2	2	93	DT
29188284	2018	2	2	26	V
30351999	2018	2	2	24	V
30690294	2019	2	3	214	T
31437754	2019	2	3	192	EB
	2019	2	3	192	E
	2019	2	3	186	V
30580112	2019	2	real world	3219	V
31732523	2019	2	1	63	VC
	2019	2	1	66	VC
30386910	2019	2	real world	139	D
	2019	2	real world	698	V
31876308	2019	2	real world	50	DT
31171444	2019	2	2	35	DT
32914022	2019	2	2	62	V
31901705	2020	2	3	192	EB
	2020	2	3	192	E
	2020	2	3	186	V
32822286	2020	2	3	201	B
32234665	2020	2	2	24	T
32534242	2020	2	2	77	DT
32818466	2020	2	2	43	DT
33020646	2020	2	2	104	DT
	2020	2	2	101	DT
33361337	2020	2	2	60	DT
32699976	2020	2	real world	112	DT
31895752	2020	1	-	271	DT
32534646	2020	2	3	281	VC
32758030	2020	2	3	35	DT

Study PMID	Year	Study type (1= retrospective 2=prospective)	Study phase (1,2,3,4, real world)	Study size	Drug
32875931	2020	1	-	62	V
34158360	2021	2	3	248	V
	2021	2	3	247	VC
34455067	2021	2	2	93	DT
33637626	2021	2	2	50	B

Table S2-Evaluation of the bias risk according to Risk Of Bias In Non-randomized Studies of Interventions guidelines (ROBINS I). (Sterne JAC, Higgins JPT, Elbers RG, Reeves BC and the development group for ROBINS-I. *"Risk Of Bias In Non-randomized Studies of Interventions (ROBINS-I): detailed guidance"*. Updated 12 October 2016. http://www.riskofbias.info

BIAS DUE TO CONFOUNDING

Study PMID	Year	1.1	1.2	1.3	1.4	1.5	1.6	1.7	1.8	Bias level
21639808	2011	N	NA	NA	NA	NA	NA	NA	NA	Low
22356324	2012	N	NA	NA	NA	NA	NA	NA	NA	Low
22663011	2012	N	NA	NA	NA	NA	NA	NA	NA	Low
22735384	2012	N	NA	NA	NA	NA	NA	NA	NA	Low
23051966	2012	N	NA	NA	NA	NA	NA	NA	NA	Low
22805291	2012	N	NA	NA	NA	NA	NA	NA	NA	Low
23020132	2012	N	NA	NA	NA	NA	NA	NA	NA	Low
22805292	2012	N	NA	NA	NA	NA	NA	NA	NA	Low
23248257	2013	PN	NA	NA	NA	NA	NA	NA	NA	Low
23406731	2013	PN	NA	NA	NA	NA	NA	NA	NA	Low
23414587	2013	PN	NA	NA	NA	NA	NA	NA	NA	Low
23918947	2013	PN	NA	NA	NA	NA	NA	NA	NA	Low
24295639	2014	N	NA	NA	NA	NA	NA	NA	NA	Low
24445759	2014	N	NA	NA	NA	NA	NA	NA	NA	Low
24508103	2014	N	NA	NA	NA	NA	NA	NA	NA	Low
24241686	2014	PN	NA	NA	NA	NA	NA	NA	NA	Low
24582505	2014	PN	NA	NA	NA	NA	NA	NA	NA	Low
25037139	2014	N	NA	NA	NA	NA	NA	NA	NA	Low
25265492	2014	N	NA	NA	NA	NA	NA	NA	NA	Low
25265494	2014	N	NA	NA	NA	NA	NA	NA	NA	Low
25287827	2014	N	NA	NA	NA	NA	NA	NA	NA	Low
25399551	2015	N	NA	NA	NA	NA	NA	NA	NA	Low
24661317	2015	PN	NA	NA	NA	NA	NA	NA	NA	Low
25952781	2015	PN	NA	NA	NA	NA	NA	NA	NA	Low
26557775	2015	PN	NA	NA	NA	NA	NA	NA	NA	Low
25722381	2015	PN	NA	NA	NA	NA	NA	NA	NA	Low
25956405	2015	N	NA	NA	NA	NA	NA	NA	NA	Low
26037941	2015	N	NA	NA	NA	NA	NA	NA	NA	Low
26287849	2015	PN	NA	NA	NA	NA	NA	NA	NA	Low
26352686	2015	N	NA	NA	NA	NA	NA	NA	NA	Low
26392102	2015	N	NA	NA	NA	NA	NA	NA	NA	Low
26109403	2015	PN	NA	NA	NA	NA	NA	NA	NA	Low
26460303	2015	PN	NA	NA	NA	NA	NA	NA	NA	Low
26208946	2016	PN	NA	NA	NA	NA	NA	NA	NA	Low
26981153	2016	PN	NA	NA	NA	NA	NA	NA	NA	Low
26983408	2016	PN	NA	NA	NA	NA	NA	NA	NA	Low
**Study PMID**	**Year**	**1.1**	**1.2**	**1.3**	**1.4**	**1.5**	**1.6**	**1.7**	**1.8**	**Bias level**

27080216	2016	PN	NA	NA	NA	NA	NA	NA	NA	Low
27071922	2016	N	NA	NA	NA	NA	NA	NA	NA	Low
27283860	2016	PN	NA	NA	NA	NA	NA	NA	NA	Low
27460442	2016	PN	NA	NA	NA	NA	NA	NA	NA	Low
27470608	2016	PN	NA	NA	NA	NA	NA	NA	NA	Low
27480103	2016	N	NA	NA	NA	NA	NA	NA	NA	Low
28151720	2017	PN	NA	NA	NA	NA	NA	NA	NA	Low
28152546	2017	N	NA	NA	NA	NA	NA	NA	NA	Low
27993793	2017	PN	NA	NA	NA	NA	NA	NA	NA	Low
28284557	2017	N	NA	NA	NA	NA	NA	NA	NA	Low
28444112	2017	N	NA	NA	NA	NA	NA	NA	NA	Low
28268064	2017	PN	NA	NA	NA	NA	NA	NA	NA	Low
28501764	2017	PN	NA	NA	NA	NA	NA	NA	NA	Low
28592387	2017	PN	NA	NA	NA	NA	NA	NA	NA	Low
28567600	2017	PN	NA	NA	NA	NA	NA	NA	NA	Low
28611198	2017	N	NA	NA	NA	NA	NA	NA	NA	Low
28475671	2017	PN	NA	NA	NA	NA	NA	NA	NA	Low
28891408	2017	N	NA	NA	NA	NA	NA	NA	NA	Low
28919011	2017	PN	NA	NA	NA	NA	NA	NA	NA	Low
28963614	2017	PN	NA	NA	NA	NA	NA	NA	NA	Low
29285228	2017	PN	NA	NA	NA	NA	NA	NA	NA	Low
29076950	2017	PN	NA	NA	NA	NA	NA	NA	NA	Low
29384960	2017	PN	NA	NA	NA	NA	NA	NA	NA	Low
29121415	2017	PN	NA	NA	NA	NA	NA	NA	NA	Low
29477665	2018	N	NA	NA	NA	NA	NA	NA	NA	Low
29188284	2018	PN	NA	NA	NA	NA	NA	NA	NA	Low
29573941	2018	N	NA	NA	NA	NA	NA	NA	NA	Low
29724167	2018	N	NA	NA	NA	NA	NA	NA	NA	Low
29785570	2018	N	NA	NA	NA	NA	NA	NA	NA	Low
30219628	2018	N	NA	NA	NA	NA	NA	NA	NA	Low
30081673	2018	PN	NA	NA	NA	NA	NA	NA	NA	Low
29438093	2018	PN	NA	NA	NA	NA	NA	NA	NA	Low
30343620	2018	N	NA	NA	NA	NA	NA	NA	NA	Low
30351999	2018	N	NA	NA	NA	NA	NA	NA	NA	Low
30468696	2019	PN	NA	NA	NA	NA	NA	NA	NA	Low
30580112	2019	PN	NA	NA	NA	NA	NA	NA	NA	Low
30690294	2019	N	NA	NA	NA	NA	NA	NA	NA	Low
31171444	2019	PN	NA	NA	NA	NA	NA	NA	NA	Low
32914022	2019	PN	NA	NA	NA	NA	NA	NA	NA	Low
31437754	2019	N	NA	NA	NA	NA	NA	NA	NA	Low
31732523	2019	N	NA	NA	NA	NA	NA	NA	NA	Low
30386910	2019	N	NA	NA	NA	NA	NA	NA	NA	Low
**Study PMID**	**Year**	**1.1**	**1.2**	**1.3**	**1.4**	**1.5**	**1.6**	**1.7**	**1.8**	**Bias level**

31876308	2019	PN	NA	NA	NA	NA	NA	NA	NA	Low
31901705	2020	N	NA	NA	NA	NA	NA	NA	NA	Low
32234665	2020	PN	NA	NA	NA	NA	NA	NA	NA	Low
31895752	2020	PN	NA	NA	NA	NA	NA	NA	NA	Low
32534242	2020	PN	NA	NA	NA	NA	NA	NA	NA	Low
32534646	2020	PN	NA	NA	NA	NA	NA	NA	NA	Low
32818466	2020	PN	NA	NA	NA	NA	NA	NA	NA	Low
32822286	2020	N	NA	NA	NA	NA	NA	NA	NA	Low
33020646	2020	PN	NA	NA	NA	NA	NA	NA	NA	Low
32758030	2020	PN	NA	NA	NA	NA	NA	NA	NA	Low
32875931	2020	PN	NA	NA	NA	NA	NA	NA	NA	Low
32699976	2020	PN	NA	NA	NA	NA	NA	NA	NA	Low
33361337	2020	PN	NA	NA	NA	NA	NA	NA	NA	Low
33637626	2021	N	NA	NA	NA	NA	NA	NA	NA	Low
34455067	2021	PN	NA	NA	NA	NA	NA	NA	NA	Low
34243078	2021	N	NA	NA	NA	NA	NA	NA	NA	Low
34158360	2021	N	NA	NA	NA	NA	NA	NA	NA	Low

BIAS IN SELECTION OF PARTICIPANTS INTO THE STUDY

Study PMID	Year	2.1	2.2	2.3	2.4	2.5	Bias level
21639808	2011	N	NA	NA	Y	NA	Low
22356324	2012	N	NA	NA	PY	NA	Low
22663011	2012	N	NA	NA	PY	NA	Low
22735384	2012	N	NA	NA	Y	NA	Low
23051966	2012	N	NA	NA	Y	NA	Low
22805291	2012	PN	NA	NA	PY	NA	Low
23020132	2012	N	NA	NA	Y	NA	Low
22805292	2012	PN	NA	NA	PY	NA	Low
23248257	2013	N	NA	NA	Y	NA	Low
23406731	2013	N	NA	NA	Y	NA	Low
23414587	2013	PN	NA	NA	PY	NA	Low
23918947	2013	PN	NA	NA	PY	NA	Low
24295639	2014	N	NA	NA	Y	NA	Low
24445759	2014	N	NA	NA	PY	NA	Low
24508103	2014	N	NA	NA	Y	NA	Low
24241686	2014	N	NA	NA	Y	NA	Low
24582505	2014	N	NA	NA	PY	NA	Low
25037139	2014	PN	NA	NA	PY	NA	Low
25265492	2014	N	NA	NA	Y	NA	Low
25265494	2014	N	NA	NA	Y	NA	Low
25287827	2014	N	NA	NA	Y	NA	Low
25399551	2015	N	NA	NA	Y	NA	Low
24661317	2015	N	NA	NA	Y	NA	Low
25952781	2015	N	NA	NA	Y	NA	Low
26557775	2015	N	NA	NA	Y	NA	Low
25722381	2015	PN	NA	NA	PY	NA	Low
25956405	2015	N	NA	NA	Y	NA	Low
26037941	2015	N	NA	NA	PY	NA	Low
26287849	2015	N	NA	NA	Y	NA	Low
26352686	2015	PN	NA	NA	PY	NA	Low
26392102	2015	N	NA	NA	PY	NA	Low
26109403	2015	PN	NA	NA	Y	NA	Low
26460303	2015	PN	NA	NA	PY	NA	Low
26208946	2016	N	NA	NA	PY	NA	Low
26981153	2016	N	NA	NA	Y	NA	Low
26983408	2016	PN	NA	NA	PY	NA	Low
27080216	2016	PN	NA	NA	PY	NA	Low
27071922	2016	PN	NA	NA	PY	NA	Low
27283860	2016	PN	NA	NA	PY	NA	Low
27460442	2016	PN	NA	NA	PY	NA	Low

27470608	2016	N	NA	NA	Y	NA	Low
27480103	2016	N	NA	NA	Y	NA	Low
28151720	2017	PN	NA	NA	PY	NA	Low
28152546	2017	PN	NA	NA	PY	NA	Low
27993793	2017	PN	NA	NA	PY	NA	Low
28284557	2017	N	NA	NA	Y	NA	Low
28444112	2017	N	NA	NA	Y	NA	Low
28268064	2017	PN	NA	NA	PY	NA	Low
28501764	2017	N	NA	NA	Y	NA	Low
28592387	2017	PN	NA	NA	PY	NA	Low
28567600	2017	N	NA	NA	Y	NA	Low
28611198	2017	PN	NA	NA	PY	NA	Low
28475671	2017	N	NA	NA	Y	NA	Low
28891408	2017	N	NA	NA	PY	NA	Low
28919011	2017	PN	NA	NA	PY	NA	Low
28963614	2017	N	NA	NA	Y	NA	Low
29285228	2017	PN	NA	NA	PY	NA	Low
29076950	2017	PN	NA	NA	PY	NA	Low
29384960	2017	N	NA	NA	Y	NA	Low
29121415	2017	PN	NA	NA	PY	NA	Low
29477665	2018	N	NA	NA	Y	NA	Low
29188284	2018	PN	NA	NA	PY	NA	Low
29573941	2018	N	NA	NA	Y	NA	Low
29724167	2018	PN	NA	NA	PY	NA	Low
29785570	2018	PN	NA	NA	PY	NA	Low
30219628	2018	N	NA	NA	Y	NA	Low
30081673	2018	N	NA	NA	Y	NA	Low
29438093	2018	PN	NA	NA	PY	NA	Low
30343620	2018	N	NA	NA	Y	NA	Low
30351999	2018	PN	NA	NA	PY	NA	Low
30468696	2019	N	NA	NA	Y	NA	Low
30580112	2019	N	NA	NA	PY	NA	Low
30690294	2019	N	NA	NA	Y	NA	Low
31171444	2019	PN	NA	NA	PY	NA	Low
32914022	2019	PN	NA	NA	PY	NA	Low
31437754	2019	N	NA	NA	Y	NA	Low
31732523	2019	PN	NA	NA	PY	NA	Low
30386910	2019	N	NA	NA	PY	NA	Low
31876308	2019	N	NA	NA	Y	NA	Low
31901705	2020	N	NA	NA	Y	NA	Low
32234665	2020	PN	NA	NA	PY	NA	Low
31895752	2020	PN	NA	NA	PY	NA	Low
**Study PMID**	**Year**	**2.1**	**2.2**	**2.3**	**2.4**	**2.5**	**Bias level**

32534242	2020	PN	NA	NA	PY	NA	Low
32534646	2020	PN	NA	NA	PY	NA	Low
32818466	2020	PN	NA	NA	PY	NA	Low
32822286	2020	N	NA	NA	Y	NA	Low
33020646	2020	PN	NA	NA	PY	NA	Low
32758030	2020	PN	NA	NA	PY	NA	Low
32875931	2020	PN	NA	NA	PY	NA	Low
32699976	2020	N	NA	NA	Y	NA	Low
33361337	2020	PN	NA	NA	PY	NA	Low
33637626	2021	PN	NA	NA	PY	NA	Low
34455067	2021	PN	NA	NA	PY	NA	Low
34243078	2021	N	NA	NA	Y	NA	Low
34158360	2021	N	NA	NA	Y	NA	Low

BIAS IN CLASSIFICATIONS OF INTERVENTIONS

Study PMID	Year	3.1	3.2	3.3	Bias level
21639808	2011	Y	Y	N	Low
22356324	2012	NI	NI	PN	Low
22663011	2012	Y	Y	N	Low
22735384	2012	Y	Y	N	Low
23051966	2012	Y	Y	N	Low
22805291	2012	Y	Y	PN	Low
23020132	2012	Y	Y	N	Low
22805292	2012	PY	Y	PN	Low
23248257	2013	Y	Y	N	Low
23406731	2013	Y	Y	N	Low
23414587	2013	PY	PY	PN	Low
23918947	2013	PY	PY	PN	Low
24295639	2014	Y	Y	N	Low
24445759	2014	NI	NI	PN	Low
24508103	2014	Y	Y	N	Low
24241686	2014	Y	Y	PN	Low
24582505	2014	Y	Y	PN	Low
25037139	2014	Y	Y	PN	Low
25265492	2014	Y	Y	N	Low
25265494	2014	Y	Y	N	Low
25287827	2014	Y	Y	PN	Low
25399551	2015	Y	Y	N	Low
24661317	2015	NI	NI	PN	Low
25952781	2015	Y	Y	PN	Low
26557775	2015	NI	NI	PN	Low
25722381	2015	Y	Y	PN	Low
25956405	2015	Y	Y	PN	Low
26037941	2015	Y	Y	N	Low
26287849	2015	NI	NI	PN	Low
26352686	2015	PY	PY	PN	Low
26392102	2015	PY	PY	PN	Low
26109403	2015	Y	PY	PN	Low
26460303	2015	PY	PY	PN	Low
26208946	2016	PY	Y	PN	Low
26981153	2016	PY	Y	PN	Low
26983408	2016	PY	PY	PN	Low
27080216	2016	PY	PY	PN	Low
27071922	2016	PY	PY	PN	Low
27283860	2016	PY	PY	PN	Low

27460442	2016	PY	PY	PN	Low
27470608	2016	Y	Y	PN	Low
27480103	2016	Y	Y	N	Low
28151720	2017	NI	PY	PN	Low
28152546	2017	Y	Y	PN	Low
27993793	2017	PY	PY	PN	Low
28284557	2017	Y	Y	N	Low
28444112	2017	Y	Y	N	Low
28268064	2017	PY	PY	PN	Low
28501764	2017	Y	Y	N	Low
28592387	2017	PY	PY	PN	Low
28567600	2017	Y	Y	PN	Low
28611198	2017	Y	Y	PN	Low
28475671	2017	Y	Y	PN	Low
28891408	2017	NI	Y	N	Low
28919011	2017	PY	PY	PN	Low
28963614	2017	Y	Y	PN	Low
29285228	2017	PY	PY	PN	Low
29076950	2017	PY	PY	PN	Low
29384960	2017	Y	Y	PN	Low
29121415	2017	PY	PY	PN	Low
29477665	2018	Y	Y	N	Low
29188284	2018	PY	PY	PN	Low
29573941	2018	Y	PY	N	Low
29724167	2018	Y	Y	PN	Low
29785570	2018	PY	Y	PN	Low
30219628	2018	Y	Y	N	Low
30081673	2018	Y	PY	N	Low
29438093	2018	PY	Y	PN	Low
30343620	2018	Y	PY	N	Low
30351999	2018	PY	PY	PN	Low
30468696	2019	Y	Y	N	Low
30580112	2019	Y	Y	PN	Low
30690294	2019	Y	Y	N	Low
31171444	2019	PY	PY	PN	Low
32914022	2019	PY	PY	PN	Low
31437754	2019	Y	Y	N	Low
31732523	2019	Y	Y	PN	Low
30386910	2019	Y	Y	PN	Low
31876308	2019	Y	Y	PN	Low
31901705	2020	Y	Y	N	Low
**Study PMID**	**Year**	**3.1**	**3.2**	**3.3**	**Bias level**
32234665	2020	PY	PY	PN	Low

31895752	2020	PY	PY	PN	Low
32534242	2020	PY	PY	PN	Low
32534646	2020	PY	PY	PN	Low
32818466	2020	PY	PY	PN	Low
32822286	2020	Y	Y	N	Low
33020646	2020	PY	PY	PN	Low
32758030	2020	PY	PY	PN	Low
32875931	2020	PY	PY	PN	Low
32699976	2020	Y	Y	N	Low
33361337	2020	PY	PY	PN	Low
33637626	2021	PY	PY	PN	Low
34455067	2021	PY	PY	PN	Low
34243078	2021	Y	Y	N	Low
34158360	2021	Y	Y	N	Low

BIAS DUE TO DEVIATIONS FROM INTENDED INTERVENTIONS

Study PMID	Year	4.1	4.2	4.3	4.4	4.5	4.6	Bias level
21639808	2011	N	NA	NI	NI	NI	NA	Low
22356324	2012	N	NA	NI	NI	NI	NA	Low
22663011	2012	N	NA	NI	NI	NI	NA	Low
22735384	2012	N	NA	NI	NI	NI	NA	Low
23051966	2012	N	NA	NA	NA	NA	NA	Low
22805291	2012	NI	NI	NI	PY	Y	NA	Low
23020132	2012	N	NA	NI	NI	NI	NA	Low
22805292	2012	NI	NI	NI	PY	Y	NA	Low
23248257	2013	PN	NA	NI	NI	NI	NA	Low
23406731	2013	NI	NA	NI	Y	Y	NA	Low
23414587	2013	PN	NA	NI	NI	NI	NA	Low
23918947	2013	PN	NA	NI	NI	NI	NA	Low
24295639	2014	N	NA	NI	NI	NI	NA	Low
24445759	2014	N	NA	NI	NI	NI	NA	Low
24508103	2014	N	NA	NI	NI	NI	NA	Low
24241686	2014	NI	NA	NI	PY	PY	NA	Low
24582505	2014	NI	NA	NI	NI	Y	NA	Low
25037139	2014	NI	NI	NI	PY	Y	NA	Low
25265492	2014	N	NA	NI	NI	NI	NA	Low
25265494	2014	N	NA	NI	NI	NI	NA	Low
25287827	2014	N	NA	PY	PY	PY	NA	Low
25399551	2015	N	NA	NI	NI	NI	NA	Low
24661317	2015	NI	NA	NI	PY	PY	NA	Low
25952781	2015	N	NA	NI	NI	NI	NA	Low
26557775	2015	NI	NA	NI	PY	PY	NA	Low
25722381	2015	PN	NA	NI	NI	NI	NI	Low
25956405	2015	NI	NA	NI	PY	PY	NA	Low
26037941	2015	N	NA	NI	NI	NI	NA	Low
26287849	2015	NI	NA	NI	PY	PY	NA	Low
26352686	2015	PN	NA	NI	NI	NI	NA	Low
26392102	2015	N	NA	PY	PY	PY	NA	Low
26109403	2015	NI	NA	NI	PY	PY	NA	Low
26460303	2015	PN	NA	NI	NI	NI	NA	Low
26208946	2016	N	NA	NI	NI	NI	NA	Low
26981153	2016	NI	NA	NI	PY	PY	NA	Low
26983408	2016	PN	NA	PY	PY	PY	NA	Low
27080216	2016	PN	NA	NI	NI	NI	NA	Low
27071922	2016	NI	NI	NI	PY	PY	NA	Low
27283860	2016	PN	NA	NI	NI	NI	NA	Low
27460442	2016	PN	NA	NI	NI	NI	NA	Low

Study PMID	Year	4.1	4.2	4.3	4.4	4.5	4.6	Bias level
27470608	2016	NI	NA	NI	PY	PY	NA	Low
27480103	2016	N	NA	NI	NI	NI	NA	Low
28151720	2017	PN	NA	NI	NI	NI	NA	Low
28152546	2017	NI	NI	NI	PY	Y	NA	Low
27993793	2017	PN	NA	NI	NI	NI	NA	Low
28284557	2017	N	NA	NI	NI	NI	NA	Low
28444112	2017	N	NA	NI	NI	NI	NA	Low
28268064	2017	PN	NA	NI	NI	NI	NA	Low
28501764	2017	NI	NA	NI	Y	Y	NA	Low
28592387	2017	PN	NA	NI	NI	NI	NA	Low
28567600	2017	NI	NA	NI	PY	PY	NA	Low
28611198	2017	NI	NI	NI	PY	Y	NA	Low
28475671	2017	NI	NA	NI	PY	PY	NA	Low
28891408	2017	N	NA	NI	NI	NI	NA	Low
28919011	2017	PN	NA	NI	NI	NI	NA	Low
28963614	2017	NI	NA	NI	PY	PY	NA	Low
29285228	2017	PN	NA	PY	PY	PY	NA	Low
29076950	2017	PN	NA	NI	NI	NI	NA	Low
29384960	2017	NI	NA	NI	PY	PY	NA	Low
29121415	2017	PN	NA	NI	NI	NI	NA	Low
29477665	2018	N	NA	NI	NI	NI	NA	Low
29188284	2018	PN	NA	NI	NI	NI	NA	Low
29573941	2018	N	NA	NI	NI	NI	NA	Low
29724167	2018	NI	NI	NI	PY	Y	NA	Low
29785570	2018	NI	NI	NI	PY	Y	NA	Low
30219628	2018	N	NA	NI	NI	NI	NA	Low
30081673	2018	NI	NA	NI	Y	Y	NA	Low
29438093	2018	NI	NA	NI	PY	Y	NA	Low
30343620	2018	N	NA	NI	NI	NI	NA	Low
30351999	2018	PN	NA	NI	NI	NI	NA	Low
30468696	2019	NI	NA	NI	Y	Y	NA	Low
30580112	2019	NI	NA	NI	NI	Y	NA	Low
30690294	2019	N	NA	NI	NI	NI	NA	Low
31171444	2019	PN	NA	NI	NI	NI	NA	Low
32914022	2019	PN	NA	NI	NI	NI	NA	Low
31437754	2019	NI	NA	NI	Y	Y	NA	Low
31732523	2019	NI	NI	NI	PY	Y	NA	Low
30386910	2019	NI	NA	NI	PY	PY	NA	Low
31876308	2019	NI	NA	NI	PY	PY	NA	Low
31901705	2020	N	NA	NI	NI	NI	NA	Low
32234665	2020	PN	NA	NI	NI	NI	NA	Low
31895752	2020	PN	NA	NI	NI	NI	NA	Low

Study PMID	Year	4.1	4.2	4.3	4.4	4.5	4.6	Bias level
32534242	2020	PN	NA	NI	NI	NI	NA	Low
32534646	2020	PN	NA	NI	NI	NI	NA	Low
32818466	2020	PN	NA	NI	NI	NI	NA	Low
32822286	2020	N	NA	NI	NI	NI	NA	Low
33020646	2020	PN	NA	NI	NI	NI	NA	Low
32758030	2020	PN	NA	NI	NI	NI	NA	Low
32875931	2020	PN	NA	NI	NI	NI	NA	Low
32699976	2020	NI	NA	NI	Y	Y	NA	Low
33361337	2020	PN	NA	NI	NI	NI	NA	Low
33637626	2021	PN	NA	NI	NI	NI	NA	Low
34455067	2021	PN	NA	NI	NI	NI	NA	Low
34243078	2021	N	NA	NI	NI	NI	NA	Low
34158360	2021	N	NA	NI	NI	NI	NA	Low

BIAS DUE TO MISSING DATA

Study PMID	Year	5.1	5.2	5.3	5.4	5.5	Bias level
21639808	2011	PY	PN	PN	NA	NA	Low
22356324	2012	PY	PN	Y	NA	Y	Low
22663011	2012	Y	N	N	NA	NA	Low
22735384	2012	Y	N	N	NA	NA	Low
23051966	2012	Y	N	N	NA	NA	Low
22805291	2012	Y	PN	PN	NA	NA	Low
23020132	2012	Y	N	N	NA	NA	Low
22805292	2012	Y	PN	PN	NA	NA	Low
23248257	2013	Y	PN	PN	NA	NA	Low
23406731	2013	NI	NI	NI	NA	NA	Low
23414587	2013	PY	PN	PN	NA	NA	Low
23918947	2013	PY	PN	PN	NA	NA	Low
24295639	2014	Y	N	N	NA	NA	Low
24445759	2014	PY	PN	Y	NA	Y	Low
24508103	2014	Y	N	N	NA	NA	Low
24241686	2014	Y	PN	PN	NA	NA	Low
24582505	2014	PY	PN	PN	NA	NA	Low
25037139	2014	Y	PN	PN	NA	NA	Low
25265492	2014	Y	N	N	NA	NA	Low
25265494	2014	Y	N	N	NA	NA	Low
25287827	2014	Y	PN	PN	NA	NA	Low
25399551	2015	Y	N	N	NA	NA	Low
24661317	2015	PY	PN	PN	NA	NA	Low
25952781	2015	PY	PN	PN	NA	NA	Low
26557775	2015	PY	PN	PN	NA	NA	Low
25722381	2015	PY	PY	NI	Y	PN	Low
25956405	2015	Y	PN	PN	NA	NA	Low
26037941	2015	PY	PN	PN	NA	NA	Low
26287849	2015	PY	PN	NI	NA	NA	Low
26352686	2015	PY	PN	PN	NA	NA	Low
26392102	2015	Y	PN	NI	NA	NA	Low
26109403	2015	PY	N	PN	NA	NA	Low
26460303	2015	PY	PN	PN	NA	NA	Low
26208946	2016	N	PN	N	NI	PY	Low
26981153	2016	Y	N	PN	NA	NA	Low
26983408	2016	PY	PN	PN	NA	NA	Low
27080216	2016	PY	PN	PN	NA	NA	Low
27071922	2016	Y	N	N	NA	NA	Low
27283860	2016	PY	PN	PN	NA	NA	Low
27460442	2016	PY	PN	PN	NA	NA	Low
27470608	2016	Y	PN	PN	NA	NA	Low

Study PMID	Year	5.1	5.2	5.3	5.4	5.5	Bias level
27480103	2016	Y	N	N	NA	NA	Low
28151720	2017	PY	PN	PN	NA	NA	Low
28152546	2017	Y	PN	PN	NA	NA	Low
27993793	2017	PY	PN	PN	NA	NA	Low
28284557	2017	PY	PN	PN	NA	NA	Low
28444112	2017	PY	PN	PN	NA	NA	Low
28268064	2017	PY	PN	PN	NA	NA	Low
28501764	2017	NI	NI	NI	NA	NA	Low
28592387	2017	PY	PN	PN	NA	NA	Low
28567600	2017	Y	PN	PN	NA	NA	Low
28611198	2017	Y	PN	PN	NA	NA	Low
28475671	2017	Y	PN	PN	NA	NA	Low
28891408	2017	PY	PN	PN	NA	NA	Low
28919011	2017	PY	PN	PN	NA	NA	Low
28963614	2017	Y	PN	PN	NA	NA	Low
29285228	2017	PY	PN	PN	NA	NA	Low
29076950	2017	PY	PN	PN	NA	NA	Low
29384960	2017	Y	PN	PN	NA	NA	Low
29121415	2017	PY	PN	PN	NA	NA	Low
29477665	2018	Y	N	N	NA	NA	Low
29188284	2018	PY	PN	PN	NA	NA	Low
29573941	2018	Y	N	N	NA	NA	Low
29724167	2018	Y	PN	PN	NA	NA	Low
29785570	2018	Y	PN	PN	NA	NA	Low
30219628	2018	Y	N	N	NA	NA	Low
30081673	2018	NI	NI	NI	NA	NA	Low
29438093	2018	Y	PN	PN	NA	NA	Low
30343620	2018	Y	N	N	NA	NA	Low
30351999	2018	PY	PN	PN	NA	NA	Low
30468696	2019	NI	NI	NI	NA	NA	Low
30580112	2019	PY	PN	PN	NA	NA	Low
30690294	2019	Y	N	N	NA	NA	Low
31171444	2019	PY	PN	PN	NA	NA	Low
32914022	2019	PY	PN	PN	NA	NA	Low
31437754	2019	NI	NI	NI	NA	NA	Low
31732523	2019	Y	PN	PN	NA	NA	Low
30386910	2019	Y	PN	PN	NA	NA	Low
31876308	2019	Y	PN	PN	NA	NA	Low
31901705	2020	Y	N	N	NA	NA	Low
32234665	2020	PY	PN	PN	NA	NA	Low
31895752	2020	PY	PN	PN	NA	NA	Low
32534242	2020	PY	PN	PN	NA	NA	Low

Study PMID	Year	5.1	5.2	5.3	5.4	5.5	Bias level
32534646	2020	PY	PN	PN	NA	NA	Low
32818466	2020	PY	PN	PN	NA	NA	Low
32822286	2020	Y	N	N	NA	NA	Low
33020646	2020	PY	PN	PN	NA	NA	Low
32758030	2020	PY	PN	PN	NA	NA	Low
32875931	2020	NI	NI	NI	NA	NA	Low
32699976	2020	NI	NI	NI	NA	NA	Low
33361337	2020	PY	PN	PN	NA	NA	Low
33637626	2021	PY	PN	PN	NA	NA	Low
34455067	2021	PY	PN	PN	NA	NA	Low
34243078	2021	PY	PN	PN	NA	NA	Low
34158360	2021	Y	N	N	NA	NA	Low

BIAS IN MEASUREMENT OF OUTCOMES

Study PMID	Year	6.1	6.2	6.3	6.4	Bias level
21639808	2011	PN	NI	PY	PN	Low
22356324	2012	PN	Y	NI	N	Low
22663011	2012	PN	PN	NI	N	Low
22735384	2012	PN	PN	NI	N	Low
23051966	2012	PN	PN	NI	N	Low
22805291	2012	NI	NI	NI	NI	Low
23020132	2012	PN	Y	PY	PN	Low
22805292	2012	NI	NI	NI	NI	Low
23248257	2013	PN	N	Y	N	Low
23406731	2013	PY	Y	Y	PN	Low
23414587	2013	PN	PY	NI	N	Low
23918947	2013	PN	PY	NI	N	Low
24295639	2014	PN	NI	NI	N	Low
24445759	2014	PN	Y	NI	N	Low
24508103	2014	PN	NI	NI	N	Low
24241686	2014	PN	NI	NI	NI	Low
24582505	2014	NI	NI	NI	NI	Low
25037139	2014	NI	NI	NI	NI	Low
25265492	2014	PN	Y	PY	PN	Low
25265494	2014	PN	Y	PY	PN	Low
25287827	2014	PN	PY	PY	PN	Low
25399551	2015	PN	Y	PY	PN	Low
24661317	2015	N	NI	NI	NI	Low
25952781	2015	PN	NI	NI	NI	Low
26557775	2015	PN	NI	NI	NI	Low
25722381	2015	PN	PY	PY	PN	Low
25956405	2015	PN	NI	NI	NI	Low
26037941	2015	N	N	PY	PN	Low
26287849	2015	PN	NI	NI	NI	Low
26352686	2015	PN	PY	NI	N	Low
26392102	2015	PN	PY	PY	PN	Low
26109403	2015	PN	NI	NI	NI	Low
26460303	2015	PN	PY	NI	N	Low
26208946	2016	PY	Y	NI	PN	Low
26981153	2016	PN	NI	NI	NI	Low
26983408	2016	PN	PY	PY	PN	Low
27080216	2016	PN	PY	NI	N	Low
27071922	2016	NI	NI	NI	NI	Low
27283860	2016	PN	PY	NI	N	Low
27460442	2016	PN	PY	NI	N	Low
27470608	2016	PN	NI	NI	NI	Low

Study PMID	Year	6.1	6.2	6.3	6.4	Bias level
27480103	2016	PN	Y	PY	PN	Low
28151720	2017	PN	PY	PY	PN	Low
28152546	2017	NI	NI	NI	NI	Low
27993793	2017	PN	PY	NI	N	Low
28284557	2017	PN	NI	PY	PN	Low
28444112	2017	PN	NI	PY	PN	Low
28268064	2017	PN	PY	NI	N	Low
28501764	2017	PY	Y	Y	PN	Low
28592387	2017	PN	PY	NI	N	Low
28567600	2017	PN	NI	NI	NI	Low
28611198	2017	NI	NI	NI	NI	Low
28475671	2017	PN	NI	NI	NI	Low
28891408	2017	N	N	PY	PN	Low
28919011	2017	PN	PY	NI	N	Low
28963614	2017	PN	NI	NI	NI	Low
29285228	2017	PN	PY	NI	N	Low
29076950	2017	PN	PY	NI	N	Low
29384960	2017	PN	NI	NI	NI	Low
29121415	2017	PN	PY	NI	N	Low
29477665	2018	PN	NI	NI	N	Low
29188284	2018	PN	PY	NI	N	Low
29573941	2018	PN	Y	PY	PN	Low
29724167	2018	NI	NI	NI	NI	Low
29785570	2018	NI	NI	NI	NI	Low
30219628	2018	PN	Y	PY	PN	Low
30081673	2018	PY	Y	Y	PN	Low
29438093	2018	PN	NI	NI	NI	Low
30343620	2018	PN	Y	PY	PN	Low
30351999	2018	PN	PY	NI	N	Low
30468696	2019	PY	Y	Y	PN	Low
30580112	2019	NI	NI	NI	NI	Low
30690294	2019	PN	Y	PY	PN	Low
31171444	2019	PN	PY	NI	N	Low
32914022	2019	PN	PY	NI	N	Low
31437754	2019	PY	Y	Y	PN	Low
31732523	2019	NI	NI	NI	NI	Low
30386910	2019	PN	NI	NI	NI	Low
31876308	2019	PN	NI	NI	NI	Low
31901705	2020	PN	Y	PY	PN	Low
32234665	2020	PN	PY	NI	N	Low
31895752	2020	PN	PY	NI	N	Low
32534242	2020	PN	PY	NI	N	Low

Study PMID	Year	6.1	6.2	6.3	6.4	Bias level
32534646	2020	PN	NI	NI	N	Low
32818466	2020	PN	PY	NI	N	Low
32822286	2020	PN	NI	NI	N	Low
33020646	2020	PN	PY	NI	N	Low
32758030	2020	PN	PY	NI	N	Low
32875931	2020	PN	PY	NI	N	Low
32699976	2020	PY	Y	Y	PN	Low
33361337	2020	PN	PY	NI	N	Low
33637626	2021	PN	PY	NI	N	Low
34455067	2021	PN	PY	NI	N	Low
34243078	2021	PN	NI	PY	PN	Low
34158360	2021	PN	Y	PY	PN	Low

BIAS IN SELECTION OF THE REPORTED RESULT

Study PMID	Year	7.1	7.2	7.3	Bias level
21639808	2011	N	N	N	Low
22356324	2012	N	N	N	Low
22663011	2012	N	N	N	Low
22735384	2012	N	N	N	Low
23051966	2012	N	N	N	Low
22805291	2012	N	N	N	Low
23020132	2012	N	N	N	Low
22805292	2012	N	N	N	Low
23248257	2013	N	N	N	Low
23406731	2013	N	NI	N	Low
23414587	2013	N	N	N	Low
23918947	2013	N	N	N	Low
24295639	2014	N	N	N	Low
24445759	2014	N	N	N	Low
24508103	2014	N	N	N	Low
24241686	2014	NI	N	PY	Low
24582505	2014	PN	PN	PN	Low
25037139	2014	N	N	N	Low
25265492	2014	N	N	N	Low
25265494	2014	N	N	N	Low
25287827	2014	N	N	N	Low
25399551	2015	N	N	N	Low
24661317	2015	NI	N	PY	Low
25952781	2015	N	PN	N	Low
26557775	2015	NI	N	PY	Low
25722381	2015	N	PN	PN	Low
25956405	2015	NI	N	PY	Low
26037941	2015	N	N	N	Low
26287849	2015	NI	N	PY	Low
26352686	2015	N	N	N	Low
26392102	2015	N	N	N	Low
26109403	2015	NI	N	PY	Low
26460303	2015	N	N	N	Low
26208946	2016	Y	PN	N	Low
26981153	2016	NI	N	PY	Low
26983408	2016	N	N	N	Low
27080216	2016	PN	N	N	Low
27071922	2016	PN	PN	PN	Low
27283860	2016	N	PN	N	Low
27460442	2016	PN	N	N	Low

Study PMID	Year	7.1	7.2	7.3	Bias level
27470608	2016	NI	N	PY	Low
27480103	2016	N	N	N	Low
28151720	2017	PN	PN	N	Low
28152546	2017	PN	N	N	Low
27993793	2017	PN	PN	PN	Low
28284557	2017	N	N	PN	Low
28444112	2017	N	N	N	Low
28268064	2017	PN	N	N	Low
28501764	2017	N	NI	N	Low
28592387	2017	PN	N	N	Low
28567600	2017	NI	N	PY	Low
28611198	2017	N	N	N	Low
28475671	2017	NI	N	PY	Low
28891408	2017	N	N	N	Low
28919011	2017	PN	N	N	Low
28963614	2017	NI	N	PY	Low
29285228	2017	PN	PN	N	Low
29076950	2017	PN	PN	N	Low
29384960	2017	NI	N	PY	Low
29121415	2017	PN	N	PN	Low
29477665	2018	N	N	N	Low
29188284	2018	PN	PN	N	Low
29573941	2018	N	PN	N	Low
29724167	2018	N	N	N	Low
29785570	2018	PN	N	N	Low
30219628	2018	N	N	N	Low
30081673	2018	N	NI	N	Low
29438093	2018	NI	N	PY	Low
30343620	2018	N	PN	N	Low
30351999	2018	PN	N	PN	Low
30468696	2019	N	NI	N	Low
30580112	2019	PN	PN	PN	Low
30690294	2019	PN	N	N	Low
31171444	2019	PN	N	PN	Low
32914022	2019	PN	N	PN	Low
31437754	2019	N	NI	N	Low
31732523	2019	N	N	N	Low
30386910	2019	NI	PN	PY	Low
31876308	2019	NI	N	PY	Low
31901705	2020	N	N	N	Low
32234665	2020	PN	N	N	Low
31895752	2020	PN	PN	PN	Low

Study PMID	Year	7.1	7.2	7.3	Bias level
32534242	2020	PN	N	N	Low
32534646	2020	PN	N	N	Low
32818466	2020	PN	N	N	Low
32822286	2020	N	N	N	Low
33020646	2020	PN	PN	N	Low
32758030	2020	PN	N	PN	Low
32875931	2020	PN	N	PN	Low
32699976	2020	N	NI	N	Low
33361337	2020	PN	PN	N	Low
33637626	2021	PN	PN	PN	Low
34455067	2021	PN	PN	PN	Low
34243078	2021	N	N	N	Low
34158360	2021	N	N	N	Low

Figure S1- Overall incidences of grade 5 adverse events according to treatment regimen.

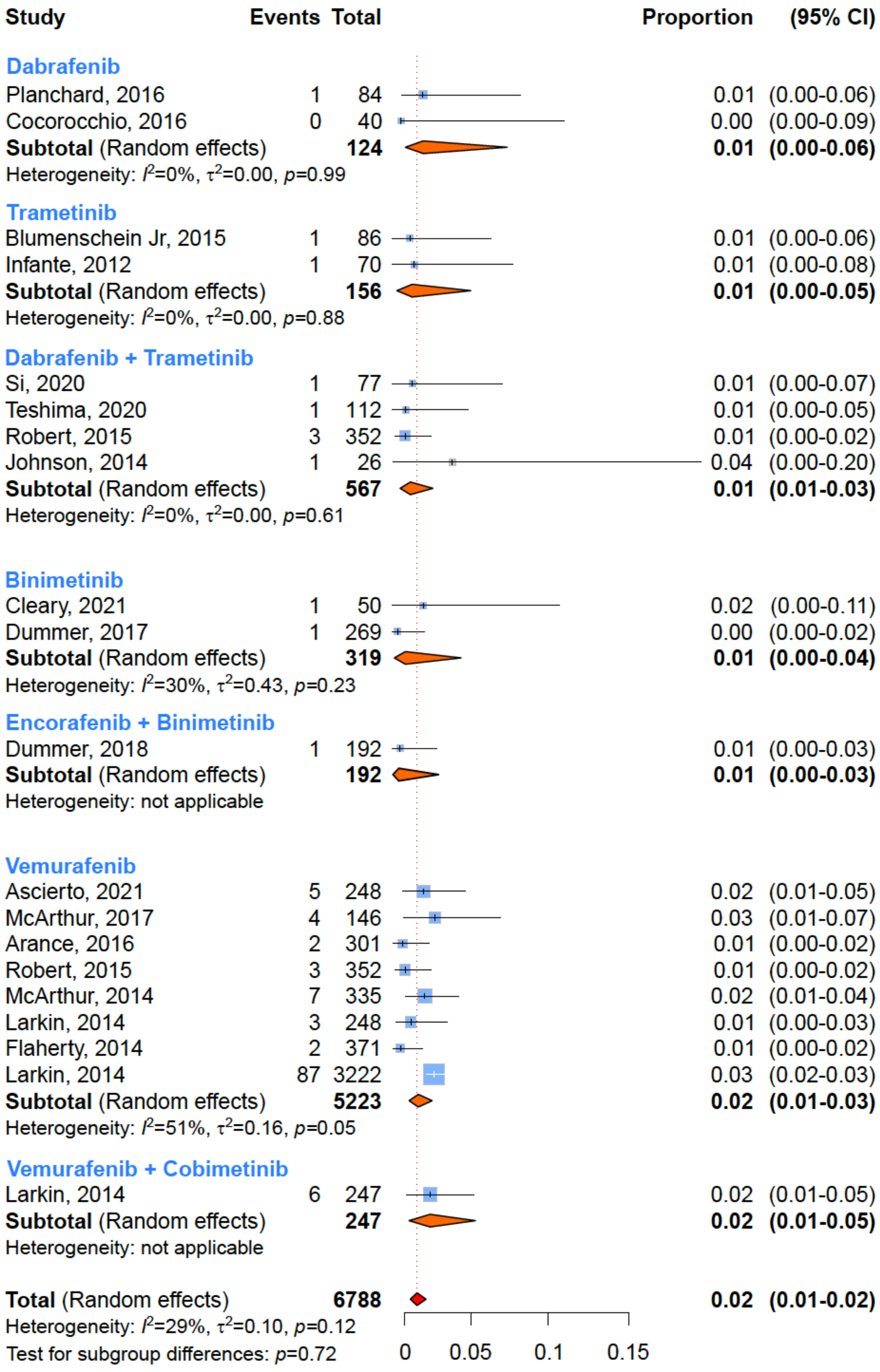

Table S3-Adverse events grade 5

DRUG	ADVERSE EVENT	N
**Vemurafenib**	Death	12
	General Physical Health Deterioration	6
	Cerebral Haemorrhage	6
	Cerebrovascular Accident	5
	Pneumonia	5
	Intracranial Tumour Haemorrhage	5
	Pulmonary Embolism	5
	Cardiac Arrest	3
	Multi-Organ Failure	3
	Sepsis	3
	Dyspnoea	3
	Cardiopulmonary Failure	2
	Myocardial Infarction	2
	Respiratory Tract Infection	2
	Completed Suicide	2
	Haemorrhagic Stroke	2
	Cerebral Ischemia	1
	Cardiac Failure	1
	Chronic Fatigue Syndrome	1
	Euthanasia	1
	Multi-Organ Disorder	1
	Sudden Cardiac Death	1
	Cerebral Haematoma	1
	Convulsion	1
	Dizziness	1
	Haemorrhage Intracranial	1
	Hydrocephalus	1
	Intracranial Pressure Increase	1
	Ischaemic Stroke	1
	Acute Respiratory Distress Syndrome	1
	Acute Respiratory Failure	1
	Chronic Obstructive Pulmonary Disease	1
	Pulmonary Oedema	1
	Pulmonary Toxicity	1
	Respiratory Failure	1
	Acute Myocardial Infarction	1
	Cardiogenic Stroke	1
	Coronary Artery Disease 2	1
	Torsade De Pointes	1
	Escherichia Sepsis	1
	Septic Shock	1
	Soft Tissue Infection	1
	Pneumocystis Jiroveci Pneumonia	1
	Anemia	1
	Agranulocytosis	1
	Disseminated Intravascular Coagulation	1
	Thrombocytopenia	1
	Fall	1

	Injury	1
	Multiple Injuries	1
	Poisoning	1
	Diverticular Perforation	1
	Gastrointestinal Necrosis	1
	Intra-Abdominal Haemorrhage	1
	Circulatory Collapse	1
	Extremity Necrosis	1
	Atelectasis	1
	Renal Colic	1
	Toxic Epidermal Necrolysis	1
	Metastases To Central Nervous System	1
	Aortic Aneurysm Rapture	1
	Pleural Infection	1
	Glioma	1
	No information	5
**Dabrafenib**	Intracranial Haemorrhage	1
**Trametinib**	Sudden Death	1
	No Information	1
**Binimetinib**	Multi-Organ Failure	1
	No information	1
**Vemurafenib + Cobimetinib**	Cardiac Arrest	1
	Coma	1
	Clostridium Difficile Colitis	1
	Myocardial Infarction	1
	Pneumonia	1
	No information	1
**Dabrafenib + Trametinib**	Cerebral Haemorrhage	2
	Brain Stem Haemorrhage	1
	Pneumoperitoneum	1
	Disease Progression	1
	Hyponatremia	1
	Neurological decompensation	1
**Encorafenib + Binimetinib**	Suicide	1
